# A Novel Missense Mutation in the ALDH13 Gene Causes Anophthalmia in Two Unrelated Iranian Consanguineous Families

**DOI:** 10.22088/acadpub.BUMS.6.2.7

**Published:** 2017-06-06

**Authors:** Mohammadreza Dehghani, Masoud Dehghan Tezerjani, Zahra Metanat, Mohammad Yahya Vahidi Mehrjardi

**Affiliations:** 1 *Medical Genetics Research Center, Shahid Sadoughi University of Medical Sciences, Yazd, Iran.*; 2 *Research and Clinical Center for Infertility, Shahid Sadoughi University of Medical Sciences, Yazd, Iran.*; 3 *Provincial Clinical Genetic Counseling Center, Zahedan University of Medical Sciences Zahedan, Iran.*; 4 *Department of Medical Genetics, Shahid Sadoughi University of Medical Sciences, Yazd, Iran.*

**Keywords:** Anophthalmia, *ALDH1A3*, consanguinity, autosomal recessive, SNP array

## Abstract

Anophthalmia or microphthalmia (A/M) is a rare group of congenital/developmental ocular malformations, characterized by absent or small eye within the orbit affecting one or both eyes. It has complex etiology with chromosomal, monogenic with high heterogeneity, and environmental causes. We performed genome SNP-array analysis followed by autozygosity mapping and sequencing in the members of two families in which three individuals are suffering from severe bilateral anophthalmia. The genetic analysis revealed a novel missense c.709G>A mutation in exon 7 of *ALDH1A3* (aldehyde dehydrogenase 1 family member A3), causing a substitution of glycine (Gly) to arginine (Arg) at residue 237. This study consolidates the importance of *ALDH1A3* gene screening in autosomal recessive anophthalmia. This variation may also be suggestive of a founder effect in the southeastern area of Iran.

Anophthalmia (OMIM 206900) is clinically characterized by absence of ocular tissue in the orbit (1). The prevalence of anophthalmia is estimated to be 1 per 30,000 live births (2). The disease can be as an isolated or syndromic form, and have genetic and environmental reasons. In cases with genetic etiology, dominant, recessive, and X-linked inheritance patterns were reported (3), and the genetic causes of severe bilateral cases (anophthalmia or severe microphthalmia; A/M) can be identified in approximately 80 percent of cases. The most important reported contributing genes are *SOX2*, *OTX2*, *FOXE3*, *PAX6*, *STRA6*, *ALDH1A3*, *RARB*, *VSX2*, *RAX *and *BMP4*, among which *SOX2* is considered as a major gene responsible for the disease (4).

In this study, we report a novel homozygous mutation in three Iranian subjects with non-syndromic bilateral anophthalmia, identified by using genome-wide SNP genotyping and autozygosity mapping in combination with direct sequencing of the coding region of *ALDH1A3* gene. 

## Cases presentation

***Family 1 (case 1 and case 2)***

**Fig. 1:**
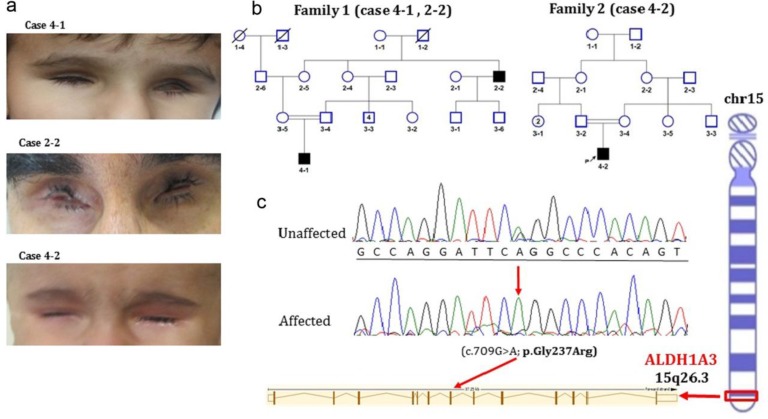
Genetic and clinical phenotype of patients. a: facial view of three affected individuals; **b**: pedigree of family 1 and 2 with three affected individuals by anophthalmia; c: sequence analysis of *ALDH1A3 *gene revealed p.Gly237Arg mutation

A 28-month-old boy (individual 4-1; fig.1b) was referred to our center because of bilateral anophthalmia. He was born as a result of first pregnancy by vaginal delivery at 36 weeks. His birth weight was 2.30 kg, length 49 cm. Her parents were healthy and consanguineous (first cousin). MRI showed that the child had bilateral anophthalmia and deep- set orbits; however, eyelids and eyelashes were normal (Fig. 1a). Absence of the retina and optic nerve function was detected by electroretinogram examination and visual evoked responses. His cognition, motor skills and growth were all normal. Cytogenetic analysis showed that he had a normal karyotype (46, XY). Familial investigation revealed the presence of the same phenotype in his 42-year-old mother's uncle (individual 2-2; Fig. 1b).

**Case 3**

A 2- year – old male (individual 4-2; Fig. 1b), was born from consanguineous parents by vaginal delivery. His birth weight and length were 2.8 kg and 51 cm, respectively. Clinical examinations revealed bilateral anophthalmia with the absence of other dysmorphic features (Fig. 1a). He had no retinal and optic nerve function. However, eyelids and eyelashes, growth and his psychomotor development were all normal.

This study was approved by the local committee of our research center and written consent was obtained. Genomic DNA was extracted from peripheral blood samples of all affected and their normal parents. We employed genome-wide single nucleotide polymorphism (SNP) array analysis, Illumina Human CytoSNP-12 V2.1 beadchip, to identify homozygous regions shared by affected individuals, and performed Sanger sequencing of A/M-associated genes located in the sizeable homozygous region.

A unique homozygosity region was shared between the two affected individuals from family 1 and defining a ~5 Mb region on chromosome 15q26. According to NCBI, this interval contained 60 annotated genes. One of the seven known non-syndromic A/M gene that localizes within the homozygous locus was *ALDH1A3*. The coding region of *ALDH1A3* was screened via direct sequencing.

A novel missense c.709G>A mutation in exon 7 of ALDH1A3 at amino acid position 237 (p.Gly237Arg) was identified in two affected individuals from the first family. The variant was absent in population variant databases including NHLBI Exome Sequencing Complete Genomics (February 2012), Project Exome Variant Server (September 2013), 1000 Genomes (May 2012), dbSNP (134–137), and Exome Aggregation Consortium (ExAC), Cambridge. After the identification of the mutation, we also checked the mutation in family 2, using F-Primer: 5’GGATGAGAAGCCCAGGTC3’ and R-primer: 5’GCCTGTCAAAGGAAAAGCTC3’. Segregation analysis demonstrated that variant segregated well with the disease within the families, and parents were all heterozygous carriers. The functional effect of the variant was predicted to be pathogenic, disease causing and damaging using in-silico software tools (SIFT, PolyPhen, Provean and MutationTaster).

## Discussion

A/M is a challenging clinical entity, as it can be caused by mutations in a big variety of genes. *ALDH1A3* gene, which has 13 exons, codes 512 amino acids that are predicted to work as a tetramer enzyme, the key enzyme that has important roles in the early eye development and neural differentiation by the formation of a retinoic-acid gradient (5, 6).

Fares-Taieet al. (2013) reported three mutations including: p.Arg89Cys, p.Ala493Pro and c.475+1G->T in *ALDH1A3* gene that causes A/M (7). Mory et al. (2014) also identified a missense mutation(c.211G4A) causing A/M in nine individuals from an inbred Muslim kindred (8). In another study, performed by Yahyavi et al. (2013), c.568A>G (p.Lys190^∗^) mutation in *ALDH1A3* was identified in two Egyptian brothers. The older brother suffered from bilateral anophthalmia, and the younger one presented anophthalmia of the right eye and severe left-sided microphthalmia, posterior coloboma, and detached retina. In addition, they found c.1165A>T (p.Lys389^∗^) mutation of *ALDH1A3* in a Hispanic girl with bilateral anophthalmia (9). The cases studied by Fares-Taie et al. (2013) showed additional clinical features such as autism and heart anomalies. However, extra ocular abnormalities are rare in patients with mutation in *ALDH1A3* gene (7)**.** Current cases such as cases studied by Mory et al. (2014) showed only eyes anomalies, making it easier for identification of the disease form and the mode of inheritance that is important for appropriate genetic counselling. According to previous studies, it has been implied that the missense mutations of *ALDH1A3* gene, leading to the loss of function of both alleles, can be lethal in humans (10). Yet, our cases with eye anomalies and with no other clinical abnormalities contradicted the hypothesis.

In conclusion, we report a novel missense mutation of *ALDH1A3* associated with autosomal recessive and congenital bilateral anophthalmia. The results indicate the importance of the mutation analysis of *ALDH1A3* gene in patients with non-syndromic bilateral anophthalmia from consanguineous families. This mutation may also be suggestive of the presence of a founder effect in the southeastern area of Iran.
